# Emotional and behavioral changes in French children during the COVID-19 pandemic: a retrospective study

**DOI:** 10.1038/s41598-023-29193-9

**Published:** 2023-02-03

**Authors:** Benjamin Landman, Alicia Cohen, Elie Khoury, Vincent Trebossen, Nesrine Bouchlaghem, Hélène Poncet-Kalifa, Eric Acquaviva, Aline Lefebvre, Richard Delorme

**Affiliations:** 1 Child and Adolescent Psychiatry Department, Robert Debré Hospital, APHP, Paris, France; 2 Human Genetics and Cognitive Functions, Institut Pasteur, Paris, France; 3 CHS Fondation Vallee, Gentilly, France

**Keywords:** Human behaviour, Anxiety

## Abstract

COVID-19 outbreak caused severe disruptions in daily life, partly due to limitations implemented to prevent the spreading. In France, it included school closures during a national lockdown, then a reopening of schools, with access depending on viral status of students and teachers. Those changes had an impact on children's mental health. We conducted an online cross-sectional study using a parental self-administered survey in December 2021 to explore the emotional and behavioral changes (EBC) during this 5th wave (W5) and retrospectively since the first one (W1) in their children and their multidimensionality with principal factor analysis (PCA) and stability analysis. Out of 4552 parent responders, 62.4% (n = 2839) noticed negative EBC during W1 and 54.1% (n = 2462) during W5 of the pandemic. Only 10.0% of the responders noticed negative EBC at W1 but not during the W5. In younger children (3–6 years old) with significant EBC, PCA revealed three main dimensions at W1 and W5: restlessness, depression and anxiety. In older children (7–13 years old), PCA showed partially similar dimensions: depression-suicidality, anxiety and withdrawal. Almost all correlations between dimensions at W1 and W5 were significantly positive. Every EBC was stable across waves, except for one. Recall bias concerning the EBC during W1 and lack of data concerning parental mental health should be taken into account. Our stability analysis found a strong correlation between dimensions at W1 and W5. Our results highlighted the impact of the COVID-19 outbreak on children's mental health and the predictive aspect of its early deterioration.

## Introduction

Childhood is a period of vulnerability for mental health, with half of all mental disorders occurring before the age of 14^1^. Since 2020, the COVID-19 pandemic has caused significant disruption in the daily lives of children with long periods of lockdown and school closures. These periods of time might have specifically affected children and adolescents, by limiting their social relationships and life experiences which are an important part of their emotional development^2^. As such, the scientific community has urged assessing the impact of the COVID crisis on mental health and well-being of children and adolescents^3^.

Worldwide, many groups reported a relatively similar impact on children's mental health co-occurring with mitigation strategies needed to limit the spread of the COVID-19 virus. Physical restrictions and social distancing were associated with an occurrence and worsening of mental health disorders in children^4^. In Spain, one study reported an increase in emotional difficulties, antisocial behaviors or conflicts with peers, specifically among those who expressed health concerns during the lockdown, or in families facing economic struggle related to the outbreak^5^. In England, 39.2% of 6–16-year-olds (y.o) experienced deterioration in mental health, and 52.5% among the 17–23 y.o. Similarly, over a quarter of the 6–10 y.o, over a third of the 11–16 y.o and over half of the 17–23 y.o reported problems with sleep on three or more nights per week during the pandemic^6^. The same epidemiological study reported a massive worsening of mental disorders in those diagnosed before the pandemic. In France, an unusually high prevalence of hyperactivity/inattention and emotional dysregulation symptoms (21.9% and 13.3%, respectively) was reported in children aged 8–9 during the first phase of the COVID-19 epidemic^7^. An independent study conducted also in France reported similar results, showing a strong correlation between emotional and hyperactivity/inattention symptoms in children, but also the role of mediators in this association such as sleep disorders, screen time exposure, parental anxiety or financial difficulties^8^. Sedentary time has risen sharply among youth during this period and may also have contributed to the decline in children's well-being^9^.

Homeschooling was similarly assessed as a potential major factor contributing to the emergence or worsening of mental health disorders in children^10^. It was associated with higher domestic conflict^11^, academic backwardness^12^, increased anxiety and suicidal behaviors^13,14^. The difficulties associated with homeschooling may have exacerbated inequalities in terms of economic, numerical and cultural disparities between social groups, acting as additional factors for worsening mental health in children^15^. Although the specific effect of homeschooling on children’s mental health was difficult to isolate, since it was included in a vast array of mitigation strategies, some studies reported that symptom intensity of mental health disorders varies in phase with the periods of homeschooling/school reopening^16,17^. These effects of school closing/reopening might be relatively modest and are heterogeneous among studies. For example, an Australian longitudinal study which explored mental health symptoms in 785 children and adolescents at different periods of the pandemic (before wave 1, during and after school closures) reported an increased level of internalizing mental health symptoms and a decrease of well-being in children and adolescents, which persisted after school-reopening, in comparison with the pre-pandemic period^18^. The authors highlighted that the absence of a control group in another region of Australia with higher rates of COVID-19 infection raise questions about the generalization of these results. Even so, a latent class analysis found that in children with the most severe difficulties, an increase in mental health difficulties took place directly from the beginning of the COVID-19 pandemic and were sustained in time^19^. After several waves of the pandemic, despite the lifting of the most constraining restrictions, significant mental health difficulties still persisted in children and adolescents with a decrease of well-being self-perception by children as reported in many countries^17,20^.

In this study, we aimed to explore the multidimensionality of mental health symptoms reported in children aged 3–12 y.o during wave 1 (W1), with onset in February 2020, and wave 5 (W5), with onset in December 2021, of the COVID-19 pandemic, as well as their stability/dynamics over time. We were similarly interested in exploring whether mask wearing was associated with reported mental health symptoms in this vulnerable population and might therefore be a factor associated with persistence of mental health impairments in children. We hypothesized that psychiatric symptoms in children gradually improved during the COVID crisis, since there was a gradual easing of mitigation measures and a less severe and worrisome collective perception of the COVID-19 pandemic. This psychiatric symptom improvement could be particularly significant in those with good mask-wearing tolerance.

## Method

### Design and participants

We conducted an online cross-sectional study using a parental self-administered survey between the 23rd and 25th of December 2021, 4 weeks after the French government officially announced the onset of the 5th COVID-19 wave as well as new strategies to prevent transmission of the new omicron-SARS-Cov2 variant (including reinforcement of social distancing at school). An invitation to fill out an online questionnaire was sent by email to subscribers of the *clepsy.fr* newsletter (a website dedicated to mental health education and parenting) and released through social media. Participants were parents of children aged between 3 and 13 y.o.

First, parents were asked about their location of residence and their child’s age and grade. Then, they were queried regarding any emotional and behavioral changes (EBC) in their children during the last 4 weeks prior to the survey (corresponding to W5) and if they had noted similar changes during W1 (February–May 2020). At any period, if changes were observed, we explored their intensity and their diversity in terms of symptoms/behaviors reported by their child.

For preschoolers (children aged 3 to 6), we explored any change regarding (1) sadness, (2) cries, (3) irritability, (4) regression in acquisitions and learning, (5) defiant and oppositional behaviors, (6) difficulty falling asleep/requiring parental presence to fall asleep, (7) solicitations not to be left alone, (8) concerns about the health of people living with the child, (9) fear of being contaminated themselves, (10) physical symptoms such as stomach aches or headaches, (11) less interest in usual activities*.* For schoolers (children aged 7–13), explored changes were: (1) sadness, (2) suicidal thoughts, (3) irritability, (4) decreased academic performance, (5) asking a lot of questions about the current crisis, (6) anxiety/fear of death, (7) difficulty falling asleep/requiring parental presence to fall asleep, (8) concerns about the health or financial status of others in the household, (9) fear of being contaminated themselves, (10) physical symptoms such as stomach aches or headaches, (11) less interest in usual activities, (12) more screen time, (13) loss of appetite.

Finally, we explored if children had complained about wearing masks during W5. If the answer was “yes”, we investigated further the intensity and the reasons for complaints. We specifically explored: (1) physical discomfort, (2) difficulty concentrating and following lessons, (3) difficulty in understanding teachers, (4) difficulty expressing themselves in class or being understood by teachers, (5) difficulty talking with classmates, (6) difficulty playing with classmates, (7) strictness about wearing the mask, (8) carelessness about wearing the mask, (9) cause of conflicts/punishments with teachers, (10) cause of conflicts with peers.

Practically, parents had to answer on a Likert scale if they noticed EBC in W5 and since W1 (No, Yes-small, Yes-moderate, Yes-strong or Yes-very strong). If the answer was yes, they had to check which EBC they noticed on a list. This EBC’s list was face-validated and based on common child behavioral and emotional responses to adversities depending on developmental stages presented by Rider et al.^21^. Our questionnaire provided this list of EBC depending on the birth age of the child and thus integrated the developmental stage described in responses to adversities from preschool to school-age. Mask complaints were explored with a list of negative changes based on qualitative evaluation in clinical settings. Easy-to-understand questions with sufficiently broad propositions were used to facilitate recall as recommended by Hipp et al.^22^.

### Data analysis

Exploratory analysis of the data was carried out using Student's t-test for continuous variables and a chi-square test for categorical ones. Logistic regression analysis was used to identify the effect of age on emotional/behavioral changes. We performed a principal component factor analysis (PCA) on mental health symptoms reported by parents in their children during the last 4 weeks prior to the survey (corresponding to W5) and during W1. We conducted a similar exploration of the symptoms associated with mask wearing. The number of factors was defined after visual exploration of the scree plot (Eigen value ≥ 1.1). To identify the items belonging to a specific factor, we observed which items had high loadings ( >*|*0.20*|*) for one factor but with low loadings ( <*|*0.10*|*) for the others.

The stability of EBC between W1 and W5 was analyzed in a 2 step-process^23^. First, we estimated for each EBC the probability of changing between W1 and W5 using McNemar's test. Second, we explored the stability of PCA dimensions across W1 and W5. Individual scores on a specific dimension at W5 were obtained by multiplying the coefficient specific to each dimension identified at W1 by the number of symptoms belonging to each dimension and endorsed by the individual at W5. We then performed a series of stepwise multiple regression models in which each dimension at W5 was the dependent variable and all the three dimensions at W1 were independent variables. Correlations of mask related dimensions and mental health related ones were explored by using a linear regression.

### Ethical approval

The research project was performed according to relevant guidelines and regulation. The protocol was submitted to the ethics review committee for research projects at the Robert Debré hospital and approval was obtained on the 30th of November 2021 under reference 2021-588. All children parents provided written informed consent at the beginning of the survey and displayed directly online.

## Results

### Participants and EBC during the pandemic

During the whole survey period, we received 4910 responses: 1427 responders were parents of preschoolers and 3483 were parents of schoolers. We removed outliers by limiting the age difference between child and school level to ± 1 year. We finally considered 4552 responses as valid (1298 preschoolers, 3254 schoolers).

The majority of parents reported EBC in their child during W1 and W5 (Table 1). Out of the 4552 participating parents, 62.4% (n = 2839) attributed negative changes in their children during W1 and 54.1% (n = 2462) during W5. Only 10.0% of the responders having observed negative changes in their child during W1, reported no similar psychiatric symptoms during W5. Conversely, only 5% of children displayed psychiatric symptoms only at W5 and none at W1. Finally, 35% of participating parents did not mention any psychiatric symptoms in their child throughout both waves.

**Table 1:** Emotional and behavioral changes along the pandemic during the 1st and the 5th wave.

	1st wave	5th wave
3–6 y.o (n)	1298	1298
Noticed changes, % (n)	57.6 (747)	49.6 (644)
Sadness	35.2 (263)	38.5 (248)
Cries	27.0 (202)	31.7 (204)
Irritability	47.3 (353)	50.3 (324)
Regression	30.0 (224)	29.8 (192)
Defiant attitude	35.7 (267)	37.6 (242)
Sleep difficulties	37.1 (277)	39.1 (252)
Solicitations	38.8 (290)	40.7 (262)
Concerns for other	28.0 (209)	36.2 (233)
Fear of contamination	24.0 (179)	24.1 (155)
Physical symptoms	24.6 (184)	29.8 (192)
Loss of interest	18.9 (141)	21.7 (140)
7–13 y.o	3254	3254
Noticed changes, % (n)	64.3 (2092)	55.9 (1818)
Sadness	44.2 (924)	45.7 (831)
Suicidal thoughts	5.4 (112)	5.6 (102)
Irritability	45.6 (954)	47.3 (860)
Decreased academic abilities	26.8 (561)	27.3 (496)
Asks a lot of questions	41.3 (863)	40.5 (736)
Anxiety/Fear of death	37.0 (773)	29.8 (542)
Sleep difficulties	35.4 (741)	33.6 (610)
Concerns for other	24.0 (503)	29.1 (529)
Fear of contamination	18.6 (390)	18.0 (327)
Physical symptoms	40.6 (849)	40.8 (742)
Loss interest	25.6 (535)	29.8 (541)
More screen time	50.0 (1045)	41.6 (756)
Loss of appetite	12.0 (251)	16.3 (297)

### Diversity of mental health symptoms in children during the pandemic

We performed a logistic regression of age on observed EBC. In preschoolers, we observed a significant age effect notably on fear of contamination (p < 0.05; Chi2 = 9.51) and physical symptoms (p < 0.05; Chi2 = 11.16) (Supplementary Fig. 1A) at W1, and on sadness (p < 0.05; Chi2 = 4.04), fear of contamination (p < 0.05, Chi2 = 9.51) and physical symptoms (p < 0.05; Chi2 = 11.58) at W5 (Supplementary Fig. 1B). For schoolers, logistic regression identified a significant age effect on EBC, for example sadness (p < 0.05; Chi2 = 4.72), suicidal thoughts (p < 0.05; Chi2 = 14.12) and academic decline (p < 0.05; Chi2 = 17.81) at W1 (Supplementary Fig. 2A), and notably suicidal thoughts (p < 0.05; Chi2 = 3.87), academic decline (p < 0.05; Chi2 = 12.18) and more screen time (p < 0.05; Chi2 = 33.34) at W5 (Supplementary Fig. 2B).

### Multidimensionality of mental health symptoms in preschoolers (children aged 3–6) during the pandemic

At W1, the PCA performed on EBC, revealed 3 main factors (variance = 42.7%): *restlessness* (defiant attitudes, irritability, solicitations, sleep difficulties), *depression (*sadness, loss of interest, cries, academic decline, physical symptoms), and a*nxiety (*fear of contamination, concerns for others) (Supplementary Table 1). We re-ran a similar analysis on symptoms reported during W5 and identified three very similar factors (variance = 45.5%) (Supplementary Table 1) suggesting a strong stability of EBC in preschoolers across waves. A multiple regression analysis showed correlations between every dimension at W1 and 5 and a cross-dimensional correlation at W1 and 5 except for *Depression* at W1 and *Anxiety* at W5. All correlations were positive except for cross-dimensions *Restlessness* and *Depression* at W1 and W5 (Table 2). At the symptom level, the Mc Nemar discordance test showed a stability in all EBC but one (*concerns for others*) (Table 3).

**Table 2:** Mc Nemar discordance test for emotional/behavioral changes.

Emotional/behavioral changes	Chi2	p value
3–6 y.o		
Sadness	0.67	0.41
Cries	2.36	0.12
Irritability	0.03	0.86
Regression	0.79	0.38
Defiant attitude	0	1.0
Sleep difficulties	0.009	0.93
Solicitations	0.24	0.62
Concerns for other	13.82	0.0002
Fear of contamination	0.01	0.91
Physical symptoms	3.31	0.07
Loss of interest	0.96	0.33
7–13 y.o		
Sadness	2.58	0.11
Suicidal thoughts	0.15	0.69
Irritability	0.02	0.88
Academic decline	2.92	0.09
Ask a lot of questions	3.97	0.05
Anxiety/fear of death	73.89	< 0.0001
Sleep difficulties	15.51	< 0.0001
Concerns for others	16.44	< 0.0001
Fear of contamination	2.41	0.12
Physical symptoms	6.78	0.009
Loss of interest	4.17	0.04
More screen time	67.76	< 0.0001
Loss of appetite	17.01	< 0.0001

**Table 3:** Fact or correlation between 1st and 5th wave.

	Restlessness (1)	Depression (1)	Anxiety (1)
3–6 y.o			
Depression (5)	0.57	− 0.05	0.07
Restlessness (5)	− 0.15	0.47	0.17
Anxiety (5)	0.08	− 0.12	0.62

	Depression–suicidality (1)	Anxiety (1)	Withdrawal (1)
7–13 y.o			
Depression—suicidality (5)	0.73	0.30	0.20
Anxiety (5)	0.22	0.61	0.18
Withdrawal (5)	0.27	0.11	0.61

In bold: correlation with p < 0.05. (1) first wave; (5) fifth wave.

### Multidimensionality of mental health symptoms in schoolers (children aged 7–13) during the pandemic

The PCA found a 3 factor model of EBC at W1 (variance = 37.4%): *depression-suicidality* (physical symptoms, sleep difficulties, sadness, irritability, loss of appetite and suicidal thoughts), *anxiety *(fear of contamination, asks many questions to others, concerns for others, anxiety/fear of death), and *withdrawal* (more screen time, loss of interest and academic decline) (Supplementary Table 2). A similar factorial structure was found at W5 (Supplementary Table 2). This also suggests a strong stability of psychiatric impairment in schoolers across waves. The multiple regression analysis showed modest positive correlations between and across dimensions at W1 and W5 (Table 3). At a symptom level, we observed significant changes for several items affecting each dimension which explained the results we reported in Table 3: *Depression-Suicidality*: sleep difficulties (Chi2 = 15.51, p < 0.05), and loss of appetite (Chi2 = 17.01, p < 0.05); *Anxiety:* anxiety/fear of death (Chi2 = 73.89, p < 0.05), concerns for others (Chi2 = 16.44, p < 0.05); *Withdrawal:* increased screen time (Chi2 = 67.76, p < 0.05), loss of interest (Chi2 = 4.17, p < 0.05).

### Mask wearing compliance correlated with mental health symptoms at wave 5

A majority of parents (60%; n = 2744) reported that their child complained about wearing their mask. Perceived physical discomfort (Chi2 = 26.3, p < 0.05), neglect of mask wearing (Chi2 = 8.85, p < 0.05), conflict with teachers (Chi2 = 22.80, p < 0.05), impact on attention span (Chi2 = 16.81, p < 0.05), and difficulty talking with peers (Chi2 = 6.01, p < 0.05) increased with age of the children. The PCA of mask compliance at W5 showed 2 factors with modest effect (variance = 24.7%): a *social dimension*, including difficulties interacting with classmates or teachers (variance = 16.9%), and a *conflict dimension*, related to conflicts with teachers or peers (variance = 7.8%) (Supplementary Table 3).

In preschoolers, the *social dimension* was associated only with the depressive dimension (R^2^ = 0.13, F = 77.09, p < 0.0001, Pearson’s r = 0.36). The *conflict dimension* was linked with the *depression*, *anxiety* and *restlessness dimensions* (R^2^ = 0.02, F = 10.11, p = 0.002, Pearson’s r = 0.14;R^2^ = 0.01, F = 7.81, p = 0.005, Pearson’s r = 0.12; R^2^ = 0.02, F = 8.74, p = 0.003, Pearson’s r = 0.13; respectively). In schoolers, the *social dimension* was associated with the *depressive, anxiety* and *withdrawal dimensions* (R^2^ = 0.09, F = 170.6, p < 0.0001, Pearson’s r = 0.29; R^2^ = 0.02, F = 36.67 p < 0.0001, Pearson’s r = 0.14; R^2^ = 0.006; R^2^ = 0.06 F = 119.51 p < 0.001, Pearson’s r = 0.25; respectively). The *conflict dimension* was associated with the *anxiety* and *withdrawal dimensions* (R^2^ = 0.006, F = 11.70 p < 0.0006, Pearson’s r = 0.08; R^2^ = 0.006; R^2^ = 0.01, F = 25.48, p < 0.0001, Pearson’s r = 0.12).

## Discussion

In our study we aimed to explore the multidimensionality of EBC reported by children aged 3–13 y.o during W1 and W5 of the COVID-19 pandemic, as well as their stability over time. In accordance with our initial hypothesis, we reported a slight improvement of EBC in children during the COVID crisis, but they remained very frequent. More than half the parents identified EBC in their child at W1 (62.4%) and W5 (54.1%). These EBC must be considered regarding the specifics of the two waves in France. During W1, schools were closed and going out in public spaces and travel were strictly controlled. At W5, schools were opened with mandatory masks inside and outside of the classroom and limited mixing in the playground and the lunchroom.

Our findings were consistent with the literature reporting persistently high levels of depression and anxiety in children and adolescents since the initial phase of the pandemic^24,25^. In Australia, when rating the mental health difficulties of their child at waves 1 and 3, a quarter of parents reported a high to very high increase in emotional and behavioral problems^26^. Exploring the mental health of 1537 children and adolescents before the pandemic and 8 weeks after the lockdown, a study showed a significant increase in their anxiety, depression, and overall psychological distress with more screen times^27^, as reported in our study. They also observed that the worsening of mental health over time was correlated with intrinsic difficulties of children facing the pandemic. Greater severity of baseline mental health symptoms was associated with a more significant increase in symptoms and greater overall psychological distress. Conversely, management of daily routines and social support were associated with less deterioration in mental health. In our study, only 10% of parents reported improvement of EBC from W1 to W5 which suggests that the other observed a stagnation or a worsening of their child’s EBC.

Our results also paralleled those in the literature which considered the impact of age on mental difficulties during the pandemic. Younger children displayed increased maladaptive behaviors since the early weeks of the pandemic with a significant increase of externalized behavior impairments (0.09; 95% CI [0.05–0.13]) and dysregulation (0.11; 95% CI [0.06–0.16])^28^. This rapid rise of disturbances may be mediated by the mental health difficulties of parents, especially in preschoolers. Parents with a high rate of stress, irritability or mood dysregulation were with children with the highest behavioral difficulties^29–31^. In older children, the pandemic negatively impacted students' achievement, especially in those from low socio-economic backgrounds^12^. They displayed an increase of social withdrawal even at home. This was consistent with the *withdrawal dimension* we reported in our study in the group of 7–13 y.o. children.

Our study suggests that EBC were globally summarized in 3 distinct dimensions. We reported a *depression dimension*, an *anxiety dimension*, and *a dimension of restlessness or withdrawal* depending on the age of the child. The youngest children had more defiant attitudes, irritability, solicitation, and sleep difficulties, and the oldest ones showed increased screen time, loss of interest and academic decline. Interestingly, and in agreement with previous results in the literature, we observed a high stability of symptoms over time: children with mental health difficulties at W1 were still affected at W5. Only 10% of those with mental health difficulties at W1 were considered by their parents to be in remission at W5. Moreover, only 5% of children who were reported by their parents as unaffected in W1 presented mental health difficulties in W5. These results suggest that the major prevention effort had to be focused on the first period of the pandemic to limit early on the number of affected children, as those would present mental health difficulties throughout the pandemic. This encourages targeting intervention strategies specifically to this group^14,32^. Those dimensions only explained part of the variance in our PCA model, the other part could be the consequence of unexplored factors. For example, literature about mental health and COVID-19 suggest strong impact of sex, socio-cultural factors and parent mental health on children emotional and behavioral difficulties^33^.

Finally, we explored the complaints expressed by children about the mandatory mask wearing during the COVID-19 pandemic. Despite an unquestionable preventive impact^34^, 60% of parents reported complaints expressed by their child. In France, in a sample of parents compliant with the mask (93.4%) but against their will (63.3%), 80.9% of children were embarrassed by the mask. The main complaints were headaches, speaking difficulties, change in mood and breathing discomfort^35^. Interestingly, anxiety symptoms were twice higher in Chinese children (aged 12–18 y.o.) who did not adhere to mask wearing practices^36^. Our PCA exposed two main dimensions in mask complaints, namely *Social* and *Conflict dimensions*. Describing a significant relationship between these two mask-related dimensions and each behavioral dimension, our results highlighted a potential link between EBC and mask wearing complaints. A factor analysis of mask wearing concerns in adults living in the USA estimated that attitudes toward public policies were influenced by three main dimensions: discomfort issues (physical and communication discomfort), external factors (overstated news about coronavirus, political beliefs, absence of mask wearing culture) and usability issues (lack of effectiveness, unnecessariness in certain, maintenance issues)^37^.

The results of our study must be considered in light of its methodological limitations. First, there was a recall bias regarding EBC reported by parents retrospectively at W1. This report could be significantly influenced by the symptoms present in the children at W5. It is worth noting that our results are consistent with another study comparing mental health in children before and after lockdown^38^ with frequent depressed and anxious mood during and after lockdown. Similarly, the emotional state of the parents responding to the questionnaires could have significantly influenced their representation of their child's mental state^29^. We did not ask for any information concerning the mental health status of parents at time of assessment which could have interfered with the report of emotional or behavioral difficulties in their children^31^. Second, we interviewed parents who were already in direct or indirect contact with our website or the information we shared on social networks about children's mental health during the pandemic. This may have led to a significant selection bias of over-representation of the mental health problems in the children of the parents we interviewed. In the same way, the structure of our questionnaire could have induced an over-representation of mental difficulties in their child. This was particularly the case with the questions on the symptoms associated with mask wearing. Some questions were seen by parents as inducing a forced choice towards a negative representation of how wearing the mask could negatively impact the child's mental health. To our knowledge, there is no validated online hetero-questionnaire to assess the EBC or mask-wearing in a large population in such an exceptional pandemic. A future validation of our questionnaire would be necessary to consider these questions. Third, we did not explore the effect of the gender of the parent or of the child for whom mental health symptoms were assessed. These questions were not included in the survey. This could also have had a significant impact on the results since the distribution of symptoms or dimensions could differ depending on gender. For example, some studies have reported that girls had a higher risk of anxiety or suicidal ideation during the COVID-19 crisis^14,39^. Finally, the absence of a control group in a different geographical place with different COVID-19 infection rate or socio-economic status could limit interpretation of our results.

## Conclusion

The rapid onset of psychiatric symptoms in the first few weeks of the pandemic and their persistence throughout the various waves underscores the need to develop new preventive strategies focused on vulnerable children rather than general population strategies. Our results tend to underline the need for rapid deployment of these prevention strategies and even better upstream of all major stresses, as in the COVID-19 pandemic. In parallel, a better understanding of these at-risk populations is essential to offer targeted primary prevention strategies.

## Supplementary Information

Supplementary Information.

## Data Availability

The datasets used and/or analysed during the current study are available from the corresponding author on reasonable request.
