# Supplementary material for: Emotional and behavioral changes in French children during the COVID-19 pandemic: a retrospective study
Source: Sci Rep. 2023 Feb 3;13:2003. doi: 10.1038/s41598-023-29193-9 (PMC9897150; doi:10.1038/s41598-023-29193-9)
Supplement: Supplementary file 1 — Supplementary Information. [file 41598_2023_29193_MOESM1_ESM.docx]

Supplementary Table 1: Varimax factor structure for emotional/behavioral changes in 3-6 y.o children

|  | **Factor loading February 2020** | | | **Factor loading November 2021** | | |
| --- | --- | --- | --- | --- | --- | --- |
|  | **Factor I Restlessness** | **Factor II Depression** | **Factor III Anxiety** | **Factor I Depression** | **Factor II Restlessneess** | **Factor III Anxiety** |
| **Emotional/Behavioral changes** |  |  |  |  |  |  |
| Sadness | 0.26 | 0.26 | **0.73** | **0.80** | -0.12 | -0.02 |
| Cries | 0.42 | **0.52** | -0.04 | **0.63** | 0.20 | -0.11 |
| Irritability | **0.66** | 0.12 | -0.15 | 0.34 | **0.42** | -0.37 |
| Regression | 0.07 | **0.50** | 0.03 | **0.48** | 0.11 | 0.08 |
| Defiant attitude | **0.66** | -0.10 | -0.21 | 0.02 | **0.57** | -0.39 |
| Sleep difficulties | **0.51** | 0.07 | 0.34 | 0.09 | **0.66** | 0.11 |
| Solicitations | **0.53** | 0.13 | 0.21 | 0.09 | **0.69** | 0.17 |
| Concerns for others | 0.06 | 0.04 | **0.77** | 0.12 | 0.13 | **0.77** |
| Fear of contamination | -0.12 | 0.03 | **0.75** | 0.05 | 0.06 | **0.78** |
| Physical symptoms | 0.23 | **0.36** | 0.21 | 0.30 | **0.35** | 0.10 |
| Loss of interest | -0.06 | **0.62** | 0.02 | **0.51** | 0.14 | 0.15 |
|  |  |  |  |  |  |  |
| **Statistics** |  |  |  |  |  |  |
| Eigenvalue | 2.12 | 1.44 | 1.14 | 2.27 | 1.59 | 1.16 |
| Variance explained | 19.3% | 32.4% | 42.8% | 20.6% | 35.1% | 45.5% |

***In bold:*** *loading values included in the factor*

Supplementary Table 2: Varimax factor structure for emotional/behavioral changes in 7-13 y.o children

|  | **Factor loading February 2020** | | | **Factor loading November 2021** | | |
| --- | --- | --- | --- | --- | --- | --- |
|  | Factor I Depression- Suicidality | Factor II Anxiety | Factor III Withdrawal | Factor I Depression-  Suicidality | Factor II Anxiety | Factor III Withdrawal |
| **Emotional/Behavioral changes** |  |  |  |  |  |  |
| Sadness | **0.51** | 0.21 | 0.14 | 0.01 | **0.51** | 0.27 |
| Suicidal thoughts | **0.41** | 0.21 | 0.14 | 0.12 | **0.48** | 0.00 |
| Irritability | **0.47** | -0.15 | 0.20 | -0.21 | **0.39** | 0.38 |
| Academic decline | 0.33 | -0.07 | **0.47** | -0.01 | 0.23 | **0.49** |
| Ask a lot of questions | -0.02 | **0.64** | -0.11 | **0.63** | 0.07 | 0.10 |
| Anxiety/Fear of death | 0.28 | **0.54** | 0.02 | **0.50** | 0.49 | -0.06 |
| Sleep difficulties | **0.54** | 0.12 | 0.01 | 0.16 | **0.58** | 0.06 |
| Concerns for others | 0.14 | **0.60** | 0.04 | **0.72** | 0.07 | 0.13 |
| Fear of contamination | -0 .06 | **0.65** | 0.14 | **0.64** | 0.03 | -0.01 |
| Physical symptoms | **0.61** | -0.04 | -0.19 | 0.01 | **0.54** | 0.10 |
| Loss of interest | 0.20 | 0.06 | **0.68** | 0.12 | 0.14 | **0.74** |
| More screen time | -0.11 | 0.07 | **0.74** | 0.21 | -0.13 | **0.74** |
| Loss of appetite | **0.45** | 0.06 | 0.26 | - 0.01 | **0.34** | **0.34** |
|  |  |  |  |  |  |  |
| **Statistics** |  |  |  |  |  |  |
| Eigenvalue | 1.79 | 1.63 | 1.45 | 1.85 | 1.79 | 1.71 |
| Variance explained | 13.8% | 12.5% | 11.1% | 14.3% | 13.8% | 13.2% |

***In bold:*** *items with high loading >|0.3|*

Supplementary Table 3: Varimax factor structure for mask complaint

|  | Factor I Interaction | Factor II Conflict |
| --- | --- | --- |
| **Mask complaint** |  |  |
| Physical discomfort | 0.26 | 0.17 |
| Concentration difficulties | **0.48** | 0.22 |
| Difficulties understanding teacher | **0.59** | 0.07 |
| Difficulties expressing themselves | **0.64** | 0.11 |
| Difficulties talking with classmates | **0.61** | 0.15 |
| Difficulties playing with classmates | **0.44** | 0.12 |
| Strict about wearing the mask | 0.00 | 0.00 |
| Careless about wearing the mask | 0.03 | 0.29 |
| Conflicts with teachers/punishments | 0.23 | **0.68** |
| Conflicts with peers | 0.13 | **0.32** |
|  |  |  |
| **Statistics** |  |  |
| Eigenvalues | 1.7 | 0.78 |
| Variance explained | 16.9% | 7.8% |

***In bold*** *: items with high loading >|0.3|*

**Supplementary Figure 1: Developmental effect on psychiatric symptoms observed by parents during Waves 1 and 5.** A. in young children from 3 to 6 y.o: 1. Logistic regression of age on psychiatric symptoms during Wave 1; 2: Logistic regression of age on psychiatric symptoms during Wave 5; B. in children from 7 to 13 y.o: 1. Logistic regression of age on psychiatric symptoms during Wave 1; 2: Logistic regression of age on psychiatric symptoms during Wave 5.

**
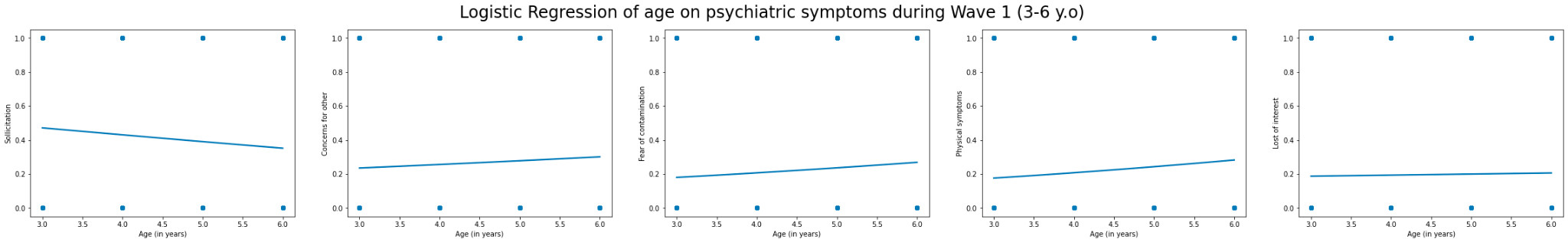

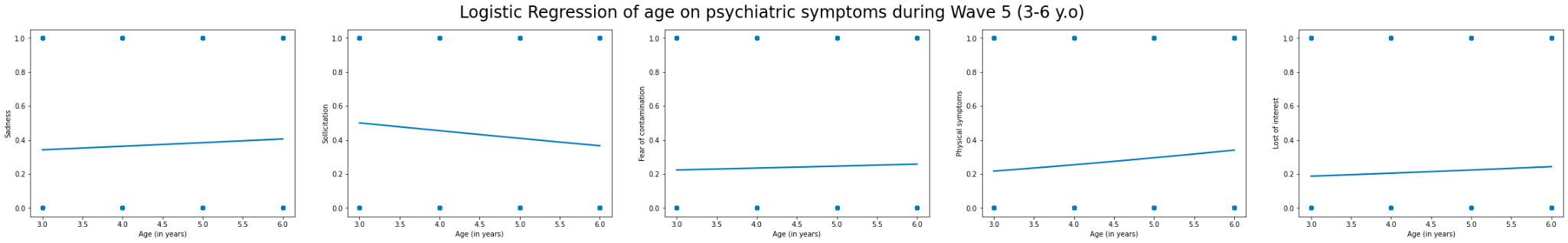
**

**
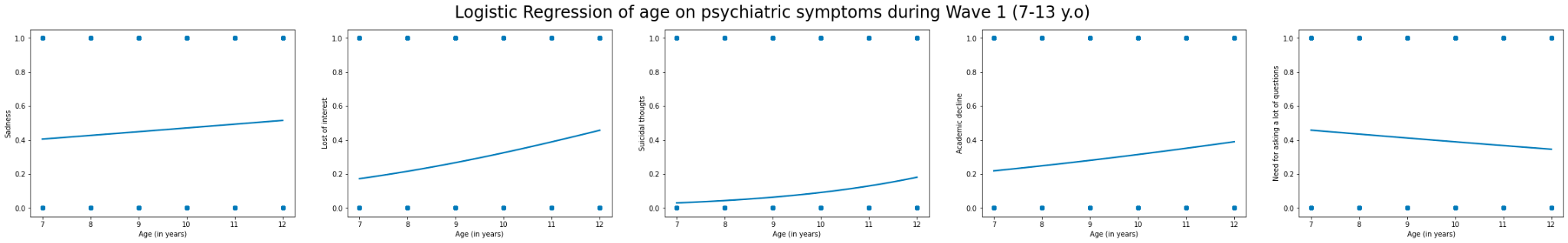
**

**
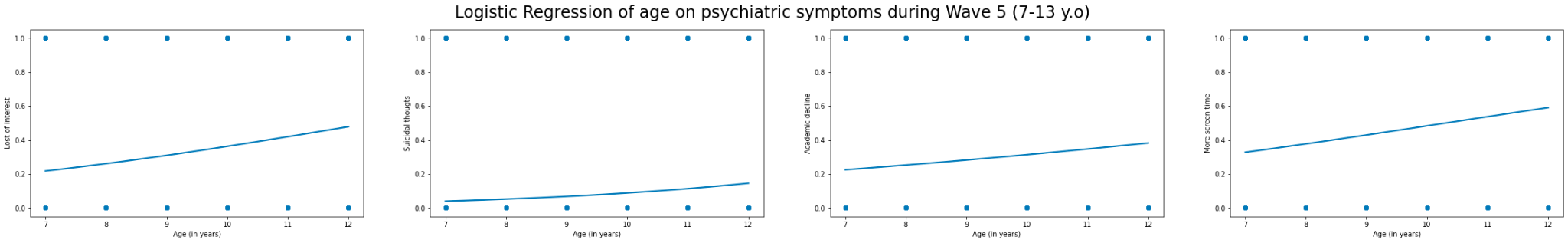
**

**Supplementary Figure 2:** Representations of the linear regression coefficient regarding the relationships between the mask waring complaints dimensions and the behavioral dimensions

A In children aged 3 to 6 y.o: representation of the linear regression coefficient obtained for Social and Conflict dimension regarding the Depression dimension (left), Anxiety dimension (middle), Restlessness (right); B. In children aged 7 to 13 y.o: representation of the linear regression coefficient obtained for Social and Conflict dimension regarding the Depression dimension (left), Anxiety dimension (middle), withdrawal (right)

**Children aged 3 to 6 y.o**

**​​
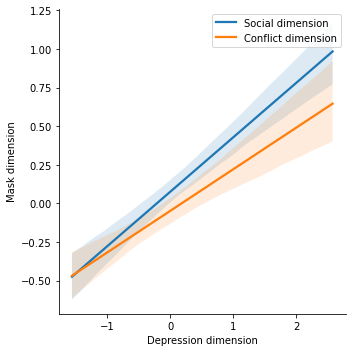

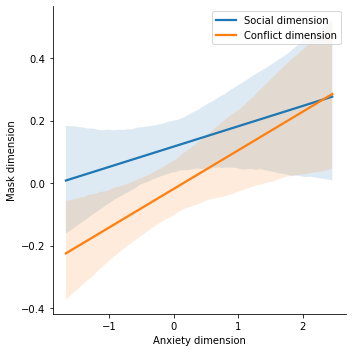

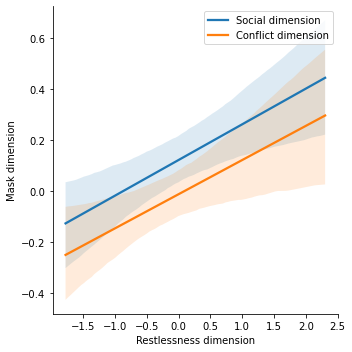
**

**Children aged 7 to 13 y.o**

**
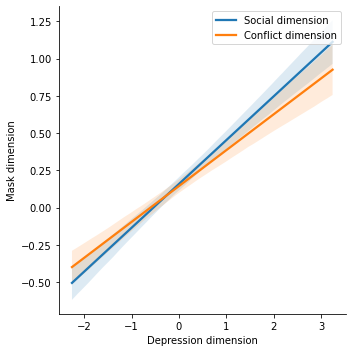

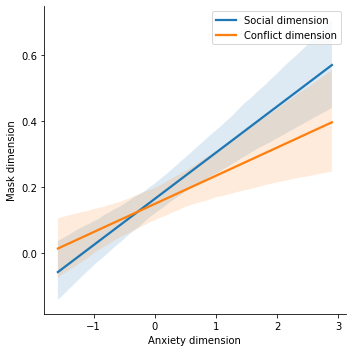

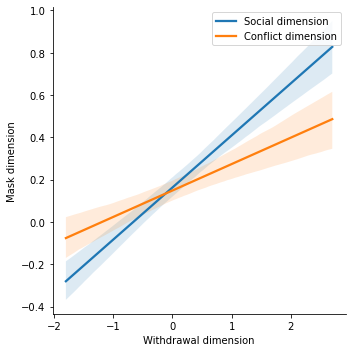
**

Supplementary table 4: Pearson correlation between mask complaints and EBC in children aged 3-6

|  | Careless about the mask | Difficulties expressing themselves | Difficulties understanding teacher | Conflicts with teachers/punishments | Concentration difficulties | Difficulties playing with classmates | Difficulties talking with classmates | Conflicts with peers |
| --- | --- | --- | --- | --- | --- | --- | --- | --- |
| Sadness | 3.96 | **19.89** | **20.33** | **31.08** | **15.93** | **11.50** | **7.69 )** | **10.87** |
| Cries | 0.84 | **16.85** | **9.31** | **24.28** | **22.006** | **11.43** | **17.18** | **6.73** |
| Irritability | 3.39) | 0.29 | **14.59** | 3.95 | **6.41** | **13.13** | **5.64** | 2.12 |
| Defient attitude | **4.99** | 0.06 | 0.68 | 0.56 | 6.88 | 1.64 | 1.004 | **7.86** |
| Loss of interest | **7.96** | **30.94** | **19.20** | **25.51** | **21.72** | **9.48** | **11.95** | **6.42** |
| Sleep difficulties | 0.39 | **4.86** | **6.18** | **7.91** | **14.61** | 1.16 | **12.22** | 2.69 |
| Physical symptoms | 2.78 | **12.06** | **6.16** | **27.46** | **5.22** | **6.43** | **4.70** | **12.97** |
| Regression | 2.28 | **42.77** | **46.96** | **19.14** | **41.51** | **6.96** | **30.81** | **10.26** |
| Solicitation | 2.04) | **7.95** | **5.59** | **18.94** | **7.78** | **6.54** | **7.12** | 3.05 |
| Fear of contamination | 1.37 | 0.71 | 2.16 | 2.99 | 0.01 | 1.33 | **6.47** | 2.17 |
| Concerns for other | 0.12 | **4.75** | 0.62 | **19.48** | 3.09 | 1.29 | **6.51** | **8.70** |

**In bold:** correlation with p<0.05

Supplementary table 5: Pearson correlation between mask complaints and EBC in children aged 7-13 year old

|  | Careless about the mask | Difficulties expressing themselves | Difficulties understanding teacher | Conflicts with teachers/punishments | Concentration difficulties | Difficulties playing with classmates | Difficulties talking with classmates | Conflicts with peers |
| --- | --- | --- | --- | --- | --- | --- | --- | --- |
| Sadness | **9.02** | **68.21** | **55.43** | **41.99** | **64.43** | **67.89** | **106.47** | **25.36** |
| Suicidal thougts | **22.29** | **10.80** | **8.49** | **30.42** | **24.61** | **14.66** | **19.66** | **47.23** |
| Irritability | **20.64** | **51.19** | **26.18** | **29.53** | **44.09** | **28.62** | **29.48** | **7.68** |
| Loss of appetite | **15.08** | **45.53** | **22.06** | **34.34** | **30.07** | **24.48** | **32.18** | **18.15** |
| Loss of interest | **10.47** | **75.98** | **47.72** | **54.88** | **73.67** | **33.48** | **67.16** | **21.55** |
| Sleep difficulties | 2.14 | **40.49** | **43.70** | **46.50** | **41.31** | **10.41** | **37.72** | **21.23** |
| Physical symptoms | **4.59** | **33.81** | **33.87** | **41.95** | **47.85** | **13.97** | **34.71** | **21.83** |
| Academic decline | **34.62** | **63.24** | **63.03** | **79.32** | **144.05** | **22.43** | **56.92** | **7.42** |
| Ask a lot of question | **14.52** | **38.47** | **23.90** | **27.48** | **29.16** | **31,78** | **50.71** | **10.95** |
| Fear of contamination | 0.58 | **8.58** | **10.78** | 2.04 | **9,17** | **9,57** | **18.61** | 3.93 |
| Anxiety/Fear of death | 2.03 | **27.83** | **33.78** | **52.71** | **34.28** | **24.98** | **35.87** | **12.63** |
| Concerns for other | 1.93 | **21.68** | **13.28** | **22.17** | **12.23** | **11.48** | **47.23** | **5.28** |
| More screen time | **22.08** | **35.28** | **17.90** | **22.19** | **26.41** | **12.05** | **13.78** | 3.82 |

**In bold:** correlation with p<0.05
